# Performance Enhancement of Ytterbium-Doped Fiber Amplifier Employing a Dual-Stage In-Band Asymmetrical Pumping

**DOI:** 10.3390/mi13091488

**Published:** 2022-09-07

**Authors:** Jawad Mirza, Salman Ghafoor, Ammar Armghan, Osama I. Elhamrawy, Laiq Jamal, Musab Magam, Sharif Iqbal Mitu Sheikh, Khurram Karim Qureshi

**Affiliations:** 1 SEECS Photonics Research Group, Islamabad 44000, Pakistan; 2 School of Electrical Engineering and Computer Science, National University of Sciences and Technology, Islamabad 44000, Pakistan; 3 Department of Electrical Engineering, College of Engineering, Jouf University, Sakaka 72388, Saudi Arabia; 4 Faculty of Engineering, Suez Canal University, Ismailia 41522, Egypt; 5 Department of Electrical Engineering and Center for Communication Systems and Sensing, King Fahd University of Petroleum and Minerals, Dhahran 31261, Saudi Arabia

**Keywords:** ytterbium-doped fiber amplifier, dual-stage pumping, small-signal gain

## Abstract

The performance of doped fiber amplifiers can be enhanced significantly with the help of multi-stage pumping technique provided that various critical parameters of pumps including their optical power and wavelength are optimized. We report the performance enhancement of a ytterbium doped fiber amplifier (YDFA) for a 1.02–1.08 μm spectral region with an optimized design based on a novel dual-stage in-band asymmetrical pumping scheme. By accurately adjusting the optical power and wavelength of pumps in both the stages, a record peak gain of around 62.5 dB and output power of 4.5 W are achieved for a signal wavelength of 1.0329 μm at an optimized length of Ytterbium-doped silica fiber and optimized doping concentration of Yb${}^{3+}$. Moreover, a minimum noise figure (NF) of 4 dB is observed for a signal wavelength of 1.0329 μm at the optimized parameters. Similarly, the effect of using high and low pump powers at the first and the second stage, respectively, on NF of the amplifier is also investigated at different values of signal powers. It is observed that the value of NF increases significantly by using high pump power at the first stage and low pump power at the second stage.

## 1. Introduction

The rare-earth elements that are widely used as dopants in the fabrication of doped-fiber amplifiers are erbium, ytterbium, praseodymium, thulium, and holmium [1]. However, the major limitation of erbium, praseodymium, thulium, and holmium is the zero emission around the 1 μm wavelength region [1]. This issue makes them useless for various applications around the 1 μm wavelength region. Thanks to ytterbium having excellent emission characteristics around the 1 μm wavelength region, it is attractive for realizing the high gain and high output power in doped-fiber amplifiers. Ytterbium-doped fiber amplifiers (YDFAs) have various attractive features that include broad gain bandwidth, high output power, high pump conversion efficiency (PCE), low thermal load, and reliable fiber geometry [2,3]. These desirable features make it ideal for various applications such as industrial manufacturing, fiber sensing, free-space optical communication, ultra-short pulse amplification, and defense applications [3,4,5]. Owing to the simple energy level structure ytterbium, many complicated atomic level processes such as excited state absorption and ion–ion interaction which normally exist in erbium-doped fiber amplifiers (EDFAs) affecting the gain dynamics do not occur in ytterbium [4]. Moreover, a wide range of pump wavelengths (0.86–1.064 μm) is available to excite the gain medium in YDFAs [4]. Therefore, research on high output power ytterbium-doping-based fiber laser and amplifier systems is progressing quickly. For instance, single transverse mode YDFAs are now capable of generating output power >2 kW and >320 W when the input signal is in continuous wave (CW) or pulsed format, respectively [6]. Similarly, it is also possible to attain high small-signal gain by incorporating a short length of fiber doped with ytterbium [4,5,6].

YDFAs have been widely researched for the past two decades. Albalawi et al. proposed a numerical model of the YDFA where the performance of the model was theoretically investigated for different pump wavelengths [2]. Liu et al. proposed a YDFA based on a double-pass two-stage configuration to study the evolution of gain and output power for different lengths of ytterbium-doped fiber (YDF) and different pump powers [5]. The maximum gain achieved in this work was 25 dB. F. He et al. proposed an optimized design of a YDFA based on cascaded pumping stages where peak small-signal gain of around 30 dB was obtained [6]. Gain characteristics of the YDFA at 1.064 μm based on double-clad and double-pump structures have been studied in [7], where a peak gain of around 45 dB was obtained. In [8], an all-fiber polarization maintaining the YDFA as pre-amplifier was proposed. The amplifier was based on a specially designed polarization maintaining YDF having a small mode field diameter and a ring-doping technique to obtain low output power when used as a pre-amplifier. M. Sajjad et al. proposed a master oscillator-power amplifier (MOPA) for the YDFA to measure small-signal gain and saturated output power [9]. The YDF employed in this work was single mode and double clad fiber, and the maximum gain and saturated output power of 25 dB and 300 mW, respectively, were obtained. J. Wu et al. investigated ion–ion interactions in Ytterbium and a proposed new model of YDFA where it was shown that the impact of ion–ion interactions is significant [10]. M. Natile et al. proposed a high-energy carrier envelope stabilized Yb-doped fiber chirped-pulse amplifier (FCPA) based on a multi-pass cell stage where nonlinear compression of the input pulses takes place. The amplifier is capable of generating optical pulses having pulse duration and energy of 96 fs and 30 μJ, respectively, at 100 kHz [11]. Y. Yu et al. demonstrated a YDFA which generates output power of 6.8 W at 0.98 μm employing a 60/130 double clad YDF and cladding-mode amplification scheme [12]. Single and double-pass YDFA configurations were studied in [13], where a double-pass configuration employing a 5 m length of YDF performs better than the single-pass by giving a maximum gain of 24.6 dB. In [14], the performance of a YDFA whose gain medium is pumped using Tandem pumping under different temperature conditions is observed. PCEs of 91.1% for 1.64 m of YDF and 81.3% for 0.82 m were obtained at room temperature and at 302 ${}^{\circ }$C, respectively. Therefore, the authors traded off 10 % PCE for a 50% reduction of YDF length. P. Yan et al. proposed a 5.4 kW output power tandem pumped YDFA in the presence of a disturbance in the refractive index of the entire length of YDF [15]. Z. Varallyay et al. proposed a numerical model of high power star-shaped bidirectional cladding pumped YDFA, generating 1 kW of output power [16]. K. K. Bobkov et al. proposed a YDFA based on tapered fiber core generating a peak power of 550 kW [17]. H. Chen et al. demonstrated a YDFA producing an output power up to 2 kW by employing distributed side-pumping and stimulated Raman scattering [18]. This novel pumping scheme consists of two sub-amplifiers which are realized by using distributed side-coupled cladding-pumps. S. Song et al. proposed an all-fiber-based technique for the measurement of phase noise to detect the variation of transient phase in a polarization maintaining YDFA based on active phase locking of an optical heterodyne detection system [19].

In view of the above discussion, we propose an optimized design to enhance the performance of the YDFA for use in various applications of optical communications in the 1.02–1.08 μm spectral range based on novel dual-stage in-band asymmetrical pumping. The pumping scheme consists of two co-propagating forward pumps. The gain medium of the first stage is pumped using a wavelength for which photon absorption is low. The gain medium of the second stage is pumped using the wavelength for which photon absorption is maximum. The pump power of the first stage is kept to a minimum compared to the second stage. Without using any optical component between the two stages, we achieved a record high peak gain of 62.5 dB and high peak output power of 4.5 W at 1.0329 μm. Furthermore, the value of NF can be scaled simply by varying the pumping wavelength and power of the first stage at the cost of a reduction in peak gain and output power of YDFA. A minimum value for NF of 4 dB has been observed for a signal wavelength of 1.070 μm. Therefore, the novel contributions of this work are summarized as follows:

- A comprehensive characterization of the YDFA in the presence of forward, backward, and bidirectional pumping is performed to obtain maximum peak gain.
- The proposed YDFA design is based on a simple and efficient dual-stage in-band asymmetrical pumping scheme.
- The proposed YDFA yields a record peak gain and output power of 62.5 dB and 4.5 W, respectively, at signal wavelength of 1.0329 μm.
- The NF and pump power conversion efficiency of 4 dB and 60.5%, respectively, is obtained at optimized parameters.
- The effect of using different pump powers at the first and second stage on the NF of the amplifier is also investigated at different values of signal powers.

We implemented the proposed design of the amplifier by using a widely known optical communication design tool called “OptiSystem” by Optiwave System Inc.,Ontario, Canada [20]. We believe that the proposed optimized design of the YDFA based on a dual-stage in-band asymmetric pumping scheme is a step forward in the development of low noise and high gain optical amplifiers. The rest of the paper is organized as follows. The detailed theoretical model of the YDFA including spectroscopic properties of Yb${}^{3+}$ in silica and rate equations are discussed in Section 2. Section 3 discusses the YDFA under different pumping schemes for performance benchmarking. Our proposed pumping scheme is discussed in Section 4, followed by its optimization in Section 5. The results are discussed in Section 6, and the effect of using high and low pump powers at first and second stage, respectively, on the NF of the amplifier is investigated in Section 7 of the paper. Finally, Section 8 concludes the paper.

## 2. Theoretical Model of the YDFA

It is necessary to understand the spectroscopic properties of Yb${}^{3+}$ in silica host to exactly model and derive the rate equations for pump and signal in YDFAs.

### 2.1. Spectroscopic Properties of Yb${}^{3+}$ in Silica Host

**Figure 1:**
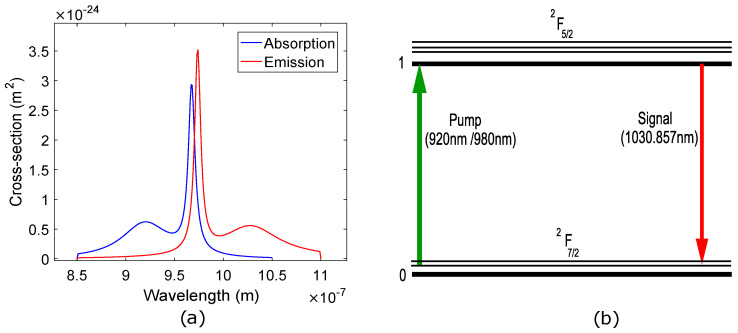
(**a**) Absorption and emission cross section of Yb${}^{3+}$; (**b**) energy level diagram of Yb${}^{3+}$.

Silica is considered to be the most commonly used ideal host material for fabrication of optical fibers for most applications. Although, some other materials such as fluoride glass are also often used for doping with Yb${}^{3+}$[4], silica is still the most widely used host material. The gain dynamics of the YDFA for a particular host material may be entirely different from other materials. Due to the suitability of silica as host material, we have also used it in our work. The spectroscopic properties of Yb${}^{3+}$ are very simple compared to other rare-earth dopants. Only two manifolds are significant in Yb${}^{3+}$ which are ^2^F_7/2_ (ground state) and ^2^F_5/2_ (excited state). These manifolds consist of various sub-energy levels, and the transitions between these levels are not fully resolved at room temperature [4]. Figure 1a shows the absorption and emission cross section of Yb${}^{3+}$ in silica glass. It may be observed that although a wide range of pump wavelengths (0.86–1.064 μm) are available to excite the Yb${}^{3+}$, maximum absorption of the pump photons takes place around 0.910 μm and 0.980 μm with emission of signal photons around 1 μm. Therefore, in this study we have employed only one transition in both of the pumping stages that is from ^2^F_7/2_→ ^2^F_5/2_ as shown in Figure 1b.

### 2.2. Rate Equations

We assume that the amplified spontaneous emission (ASE) noise does not have significant power and that the broadening in the gain medium is purely homogeneous. This results in a simplified numerical model of the YDFA. The different notations used in the following mathematical expressions are defined in Table 1. The excited and ground state populations may be expressed by the following carrier rate equations [4,6]:

(1) $$\frac{dn_{2}}{dt}=\left(R_{12}+W_{12}\right)n_{1}-\left(R_{21}+W_{21}+A_{21}\right)n_{2}$$

(2) $$\frac{dn_{1}}{dt}=-\left(R_{12}+W_{12}\right)n_{1}+\left(R_{21}+W_{21}+A_{21}\right)n_{2}$$

Under steady state conditions, the population of both the levels can be written as:

(3) $$n_{2}=\frac{R_{12}+W_{12}}{R_{12}+R_{21}+W_{12}+W_{21}+A_{21}}$$

(4) $$n_{1}=1-n_{2}$$

The transition rates between both the states may be written as [4]:

(5) $$R_{12}=\sigma_{12}^{p}\frac{I_{p}}{h\upsilon_{p}},R_{21}=\sigma_{21}^{p}\frac{I_{p}}{h\upsilon_{p}}$$

(6) $$W_{12}=\sigma_{12}^{s}\frac{I_{s}}{h\upsilon_{s}},W_{21}=\sigma_{21}^{s}\frac{I_{s}}{h\upsilon_{s}}$$

Some fraction of the pump and signal powers may propagate in the undoped cladding of the YDF [21]. Therefore, by introducing the overlap factors for the pump and signal, the propagation equations of the pump and signal at a given longitudinal position *z* along the fiber can be written as:

(7) $$\frac{dP_{p}}{dz}=\eta_{p}\left(\sigma_{21}^{p}n_{2}-\sigma_{12}^{p}n_{1}\right)n_{t}P_{p}$$

(8) $$\frac{dP_{s}}{dz}=\eta_{s}\left(\sigma_{21}^{s}n_{2}-\sigma_{12}^{s}n_{1}\right)n_{t}P_{s}$$

## 3. Characterization of the YDFA through Different Pumping Schemes for Performance Benchmarking

**Figure 2:**
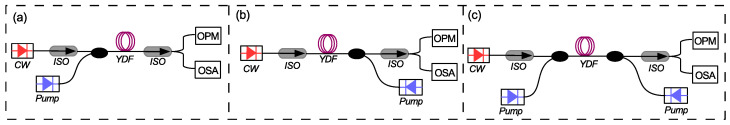
YDFA under different pumping configurations: (**a**) forward; (**b**) a backward; (**c**) bidirectional.

**Figure 3:**
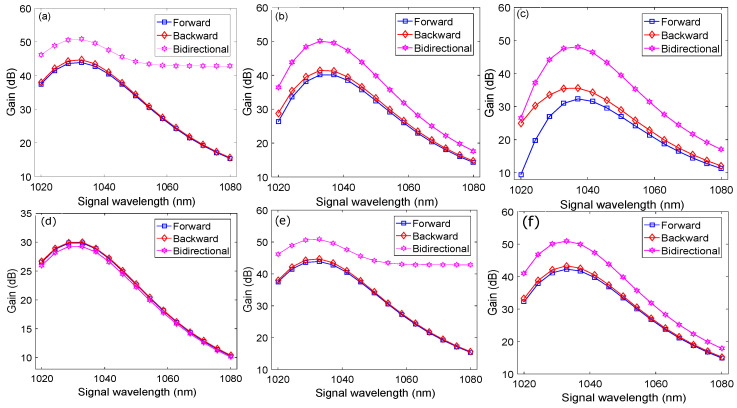
Gain optimization for performance benchmarking of the YDFA under different pumping configurations: (**a**) L = 2.5 m, $50\times 10^{24}$ m${}^{-3}$; (**b**) L = 5 m, $50\times 10^{24}$ m${}^{-3}$; (**c**) L = 7.5 m, $50\times 10^{24}$ m${}^{-3}$; (**d**) $25\times 10^{24}$ m${}^{-3}$, L = 2.5 m; (**e**) $50\times 10^{24}$ m${}^{-3}$, L = 2.5 m; (**f**) $75\times 10^{24}$ m${}^{-3}$, L = 2.5 m.

To validate the performance enhancement of the YDFA achieved through our proposed design, we first characterize the performance of the YDFA by employing conventional pumping schemes such as forward, backward, and bidirectional pumping. The length of YDF, the doping concentration of Yb${}^{3+}$, and the pumping configurations are three important factors that typically affect the performance of a doped fiber amplifier. Therefore, these factors must be tuned to achieve optimized performance of the YDFA and will be used as a benchmark to observe the performance of our proposed design. We have optimized the length of YDF, the doping concentration of Yb${}^{3+}$, and the pumping configuration with the help of signal wavelength versus gain plots. Figure 2 shows a typical YDFA where different conventional pumping configurations are used to excite the gain medium of the YDF. Figure 2a shows a forward pumping configuration where the pump andthe signal co-propagate through the YDF, Figure 2b shows backward pumping configuration in which the pump and signal counter propagate through the YDF, while Figure 2c shows a bidirectional configuration in which two pumps are used for pumping the gain medium from both ends of the YDF. By keeping the total pump power and doping concentration fixed at 5 W and $50\times 10^{24}$ m${}^{-3}$, respectively for all three cases, signal wavelength versus gain plots are obtained at 2.5 m, 5 m, and 7.5 m lengths of the YDF, as shown in Figure 3a–c, respectively. Similarly, by keeping the total pump power and optimized length of the YDF fixed at 5 W and 2.5 m, respectively, for all three cases, signal wavelength versus gain plots are obtained at doping concentrations of $25\times 10^{24}$ m${}^{-3}$, $50\times 10^{24}$ m${}^{-3}$ and $75\times 10^{24}$ m${}^{-3}$, as shown in Figure 3d–f, respectively. It may be observed from Figure 3 that through bidirectional pumping, a peak gain of 51 dB at a signal wavelength of 1.0329 μm is achieved for 2.5 m YDF that has a doping concentration of $75\times 10^{24}$ m${}^{-3}$. We consider this value of peak gain as a benchmark against which the performance of our proposed design will be compared.

## 4. Proposed Dual-Stage In-Band Asymmetrical Pumping

The concept behind our proposed pumping scheme is based on the fact that the gain and power level of the optical signal at the output of first stage plays a significant role in minimizing or maximizing the peak gain, power, and NF of the output of the second stage. Therefore, we optimize the gain and power level of the signal at the output of the first stage by employing dual-stage in-band asymmetrical pumping. It may be observed from the absorption and emission spectra of Yb${}^{3+}$ in Figure 1a that there are two pump absorption peaks. The first one is centered around 0.92 μm, while the other is centered around 0.975 μm. The absorption cross section of 0.92 μm is smaller compared to 0.975 μm. In this work, the gain medium of the first stage, which is a short piece of YDF having a length of 1 m and Yb${}^{3+}$ concentration of 50 × 10${}^{24}$ m${}^{-3}$, is excited through a pump having 1 W of power at a wavelength of 0.92 μm, where the absorption of the pump photons is lower. As a result, the gain medium experiences low population inversion and stimulated emission [22]. Consequently, a limited buildup is achieved for the gain, optical power, and ASE of the signal at the output of the first stage which is given as the input to the second stage. In the same way, the gain medium of the second stage, whose length is greater than the gain medium of the previous stage, while having the same Yb${}^{3+}$ concentration, is excited through a pump of 4 W having a wavelength of 0.98 μm, where the absorption of the pump photons is maximum. This time, the gain medium experiences maximum population inversion and stimulated emission [22], resulting in peak gain of the amplifier at the output of the second stage with limited ASE. As the buildup of the ASE of the amplifier at the output of the second stage is controlled through low power pumping of the first stage at a wavelength where the absorption of the pump photons is low, the NF of the YDFA is reduced significantly. In our optimized design of the YDFA, we have achieved enhanced performance without using any optical component between the two stages. In contrast, the authors in [23] use a passive coupler and filter between the two stages to filter out the ASE at the output of the first stage from entering into the second stage.

## 5. Proposed Optimized Design of the YDFA

**Figure 4:**
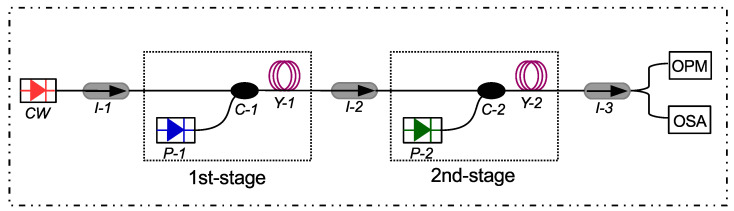
Schematic of the proposed YDFA: **CW**, continuous wave laser; **I-1/I-2/I-3**, isolators; **P-1/P-2**, pumps; **Y-1/Y-2**, YDFs; **C-1/C-2**, WDM couplers; **OSA**, optical spectrum analyzer; **OPM**, optical power meter.

Figure 4 shows the schematic of the proposed YDFA. The design consists of an input signal (CW laser); three isolators (I-1, I-2, and I-3), used to ensure unidirectional flow of the optical signal; two couplers (C-1 and C-2), used to couple the optical signal with pumps (P-1 and P-2) at both the stages; and two short pieces of YDF (Y-1 and Y-2) that serve as the gain medium. The YDFs Y-1 and Y-2 are the most critical components in the amplifier design, and their parameters are shown in Table 2. These parameters are similar to the commercially available YDF Model#YB1200-4/125 manufactured by Thorlab [24]. I-1 and I-2 in the setup are placed before Y-1 and Y-2, respectively while I-3 is placed at the output of Y-2 to suppress all possible back reflections which can disturb the stable operation of the amplifier [22,25]. Finally, an optical power meter (OPM) and an optical spectral analyzer (OSA) are used in the setup for analysis of the results.

The important parameters used in the simulation are shown in Table 2.

## 6. Results and Discussion

**Figure 5:**
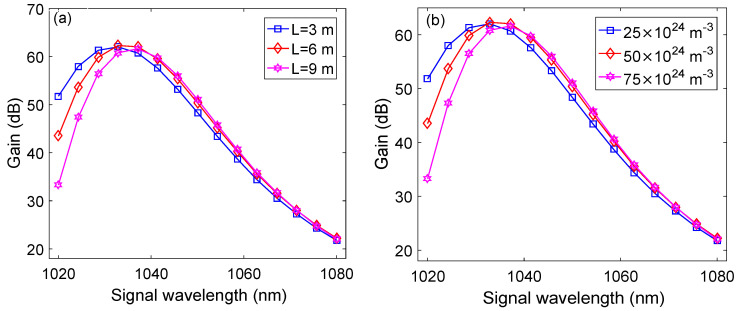
Signal wavelength versus gain plots at: (**a**) different lengths of Y-2; (**b**) different doping concentrations of Yb${}^{3+}$.

As discussed in Section 3, after optimizing the length of YDF and the doping concentration of Yb${}^{3+}$, we have obtained a peak gain of 51 dB at a signal wavelength of 1.0329 μm that is considered the benchmark against which the peak gain of our proposed YDFA will be compared. To optimize the performance of our design, the length and doping concentration of Yb${}^{3+}$ in Y-2 needs to be optimized while keeping the power of P-1 and the length and doping concentration of Y-1 fixed at 1 W, 1 m, and 50 × 10${}^{24}$ m${}^{-3}$, respectively. The peak gain of the YDFA is observed by varying the signal wavelength in the 1.02–1.08 μm range for different lengths of Y-2 (Figure 5a) and different doping concentration of Yb${}^{3+}$ in Y-2 (Figure 5b). The power of the signal and P-2 is kept at −35 dBm and 4 W. It may be observed from Figure 5a that a peak gain of around 62 dB, 62.5 dB, and 60.8 dB is observed at 3 m, 6 m, and 9 m lengths of Y-2, respectively, for the signal wavelength of 1.0329 μm. The peak gain reduces on increasing the length beyond 6 m which is due to a decrease in population inversion inside the gain medium [22]. Similarly, Figure 5b shows that a peak gain of 62.02 dB, 62.5 dB, and 60.5 dB is obtained at Yb${}^{3+}$ doping concentrations of 25 × 10${}^{24}$ m${}^{-3}$, 50 × 10${}^{24}$ m${}^{-3}$, and 75 × 10${}^{24}$ m${}^{-3}$, respectively, for a signal wavelength of 1.0329 μm at the optimized length of Y-2. Therefore, a length of 6 m and a Yb${}^{3+}$ doping concentration of 50 × 10${}^{24}$ m${}^{-3}$ gives the highest peak gain of 62.5 dB. This record peak gain of 62.5 dB is higher by 11.5 dB than the benchmark at the same conditions.

**Figure 6:**
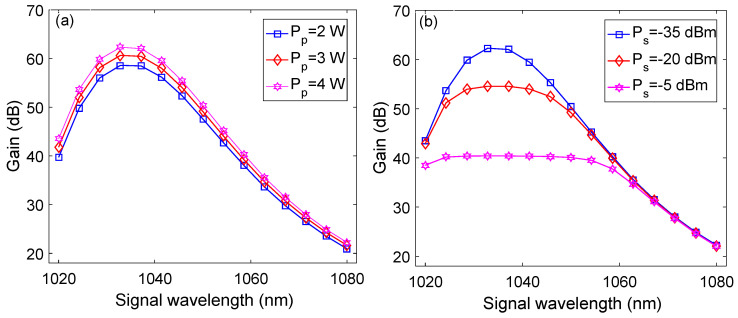
Signal wavelength versus gain plots at: (**a**) different pump powers; (**b**) different signal powers.

Figure 6 shows signal wavelength versus gain plots at different values of pump and signal powers at optimized parameters. It may be observed from Figure 6a that peak gains of around 58.6 dB, 60.6 dB, and 62.5 dB have been obtained at pump powers of 2 W, 3 W, and 4 W, respectively, for a signal wavelength of 1.0329 μm. Similarly, peak gains of 62.5 dB, 54.5 dB, and 40.4 dB have been observed for signal powers of −35 dBm, −20 dBm, and −5 dBm, respectively, and a signal wavelength of 1.0329 μm.

**Figure 7:**
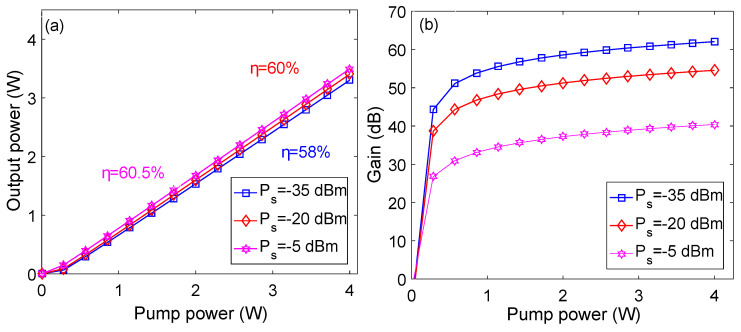
Pump power versus: (**a**) output power plots; (**b**) gain plots.

Figure 7 shows pump power versus output power and gain plots at optimized parameters at different values of signal power. It may be observed from Figure 7a that PCEs of 58.8%, 60%, and 60.5% are obtained for signal powers of −35 dBm, −20 dBm, and −5 dBm, respectively, and a signal wavelength of 1.0329 μm. As shown in Figure 7b, the gain is almost zero when pump power is 0 W at different values of signal power which is due to the fact that lasting action does not occur in the absence of pump power. The gain quickly increases on increasing the pump power for each value of signal power; however, the highest gain is achieved for the signal power of −35 dBm.

**Figure 8:**
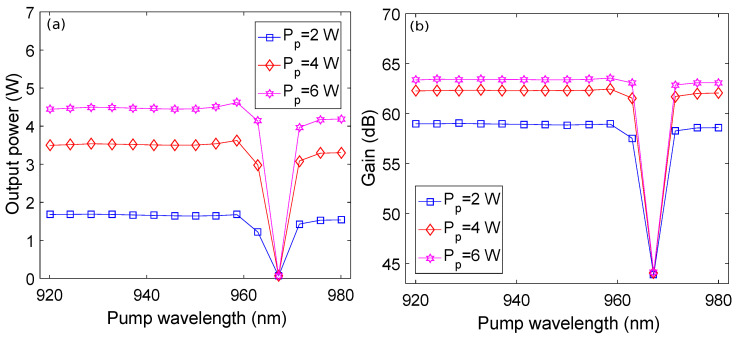
Pump wavelength versus: (**a**) output power plots; (**b**) gain plots.

Figure 8 shows the impact of a variation of the pump wavelength on the output power and gain of the amplifier for three different values of pump power at a signal wavelength of 1.0329 μm. It may be observed from Figure 8a that the output power of the amplifier remains stable around 1.75 W, 3.5 W, and 4.5 W for pump powers of 2 W, 4 W, and 6 W, respectively, while varying the pump wavelength from 0.940 μm to 0.960 μm. For the same range of pump wavelength, the gain of the amplifier is around 59 dB, 62.5 dB, and 63.5 dB for pump powers of 2 W, 4 W, and 6 W, respectively, as shown in Figure 8b. The output power and gain plots show a sharp dip around 0.965 μm for each value of pump power. On further increasing the pump wavelength beyond 0.965 μm, the plots for output power and gain approximately retain their previous pattern. This particular behavior may be understood from Figure 1a, where it can be observed that the absorption of pump photons is almost the same in the 0.940–0.960 μm wavelength range. Therefore, the output power and gain remain stable in this wavelength range for all three values of pump powers. Figure 1a shows a sharp absorption peak around 0.965 μm where absorption of pump photons is highest. We believe that the highest absorption of the pump photons around 0.965 μm at high pump powers saturates the amplifier, resulting in an interim decrease in stimulated emission. Consequently, the output power and gain of the amplifier suddenly decreases. This saturation state exists until the absorption of pump photons is reduced, bringing the output power and gain to their previous values.

**Figure 9:**
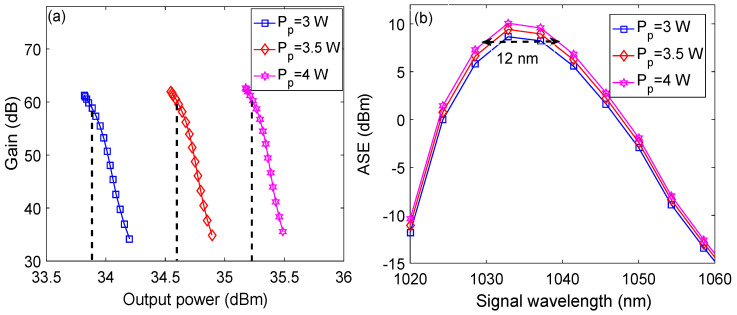
(**a**) Output power versus gain plots as a function of pump power; (**b**) signal wavelength versus ASE plots as a function of pump power.

The highest output power obtained from an optical amplifier when a seed of high power, typically around 0 dBm, is given as input to the amplifier is called 3 dB saturated output power [26]. Figure 9a,b shows the plots of the output power versus the gain and the signal wavelength versus the ASE power, respectively, as a function of pump power when the signal power is kept at −35 dBm. It may be observed from Figure 9a that 3 dB gain saturation is obtained at output powers of 33.9 dBm, 34.6 dBm, and 35.3 dBm for pump powers of 3 W, 3.5 W, and 4 W, respectively, at optimized parameters for a signal wavelength of 1.0329 μm. Similarly, a peak ASE power of 8.7 dBm is observed at a signal wavelength of 1.0329 μm for pump power of 3 W. The ASE power peaks to 9.4 dBm and 10.1 dBm for pump powers of 3.5 W and 4 W, respectively. The peak ASE power increases with the pump power because an increase in pump power not only increases the stimulated emission but also the spontaneous emission. Moreover, 3 dB bandwidth of 12 nm is obtained for pump power of 4 W, as shown by the dashed line in Figure 9b.

**Figure 10:**
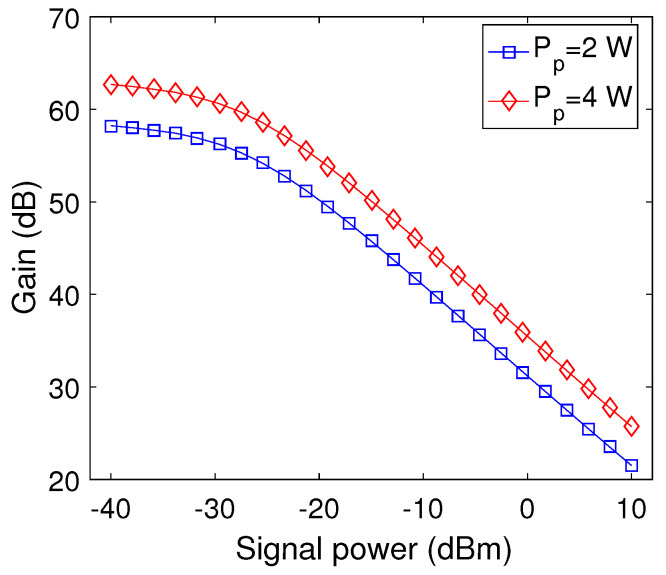
Signal power versus gain plots as a function of pump power.

To demonstrate the gain saturation, we plot the signal power versus gain as a function of pump power at optimized parameters for a signal wavelength of 1.0329 μm as shown in Figure 10. It may be observed that the gain gradually increases on decreasing the signal power up to −30 dBm from 10 dBm for both levels of pump power. On further decreasing the signal power beyond −30 dBm up to −40 dBm, the gain saturates and stops increasing further for both values of pump power. Moreover, it may also be observed that the buildup of the gain is higher at a high value of pump power.

**Figure 11:**
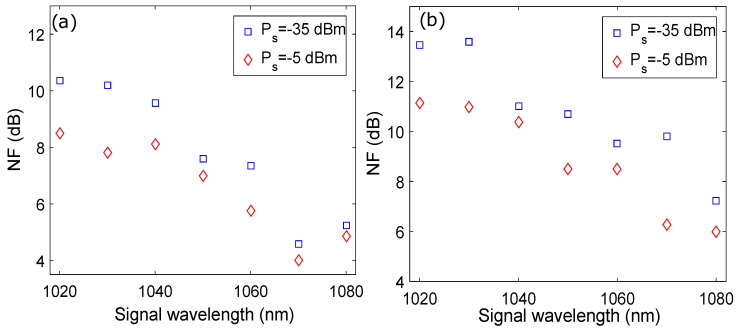
Signalwavelength versus NF plots as a function of signal power when: (**a**) $P_{1}$ = 1 W and $P_{2}$ = 4 W; (**b**) $P_{1}$ = 4 W and $P_{2}$ = 1 W.

The NF of the amplifier has been evaluated for signal wavelengths between 1.020 μm and 1.080 μm and at signal powers of −35 dBm and −5 dBm, as shown in Figure 11a. At a signal wavelength of 1.070 μm, minimum values of NF of 4.6 dB and 4 dB are observed for signal power of −35 dBm and −5 dBm, respectively. Generally, the OSNR of the amplified signal deteriorates during the process of amplification as a result of ASE that increases rapidly when the signal power is low [22]. This is the reason behind low values of NF for the 1.020 μm to 1.080 μm wavelength range for signal power of −5 dBm as compared to signal power of −35 dBm.

## 7. Effect of First-Stage Pumping on the Performance of the YDFA

To investigate the effect of using high and low pump powers at the first and second stage on the NF of the amplifier, the powers of P-1 and P-2 are chosen as 4 W and 1 W, respectively, while keeping the length and doping concentration of Yb${}^{3+}$ in Y-1 and Y-2 constant. Figure 11b shows the signal wavelength versus the NF plots as a function of signal power by choosing pump powers P-1 and P-2 as 4 W and 1 W, the length of Y-1 and Y-2 as 1 m and 6 m, respectively, and the Yb${}^{3+}$ concentration of both Y-1 and Y-2 as 50 × 10${}^{24}$ m${}^{-3}$. It may be observed from Figure 11b that the NF of the amplifier has increased compared to Figure 11a after adjusting the powers of P-1 and P-2 as 4 W and 1 W for signal powers of −35 dBm and −5 dBm, respectively. Therefore, minimum values of the NF of 10 dB and 6.3 dB are observed at a signal wavelength of 1.070 μm for signal powers of −35 dBm and −5 dBm, respectively. The reason behind this trend is that pumping Y-1 with a high power of 4 W generates an excessive amount of ASE at the output of the first stage despite using the pump wavelength of 0.92 μm, where the absorption of the pump photons is lower. The Y-2 in the second stage is pumped by a significantly lower power of 1 W compared to the first-stage pumping. Apparently, the difference in generation of ASE at the output of the second stage should not be high because maximum amplification and ASE generation have already been achieved at the first stage. However, since the pump wavelength of 0.980 μm, where the absorption of the pump photons is highest, generates more ASE than expected despite the low pump power, there is an increase in the NF of the amplifier.

Various designs of YDFAs have already been proposed in the literature as discussed in Section 1. We compare the important results of this study with previously reported studies in the literature to highlight the improvement in performance of the proposed work. Table 3 shows a detailed comparison based on important results between the proposed work and past studies. It may be observed from Table 3 that the proposed design of the YDFA shows better performance than the results of past studies [5,7,8,9,12,13,27]. A “dash” in a certain row of Table 3 represents that the information about this parameter is not provided in the particular study.

## 8. Conclusions

Performance enhancement of the YDFA has been demonstrated through optimized design for the 1.02–1.08 μm wavelength range based on a dual-stage in-band asymmetrical pumping scheme. The pumping method is based on two co-propagating pump sources where the gain medium of the first stage is excited using a wavelength for which the absorption is small, and the gain medium of the second stage is excited using a wavelength for which absorption is highest while keeping the pump power of the first stage at a minimum value compared to the second stage. A record peak gain of 62.5 dB and output power of 3.5 W have been achieved for a signal wavelength of 1.0329 μm. The NF of the amplifier has been evaluated for 1.020 μm to 1.080 μm wavelength range, and a minimum NF of 4.01 dB has been observed for a signal wavelength of 1.07 μm. The effect of interchanging the powers of the pumps of both stages on the NF of the amplifier has also been investigated. It has been suggested that certain techniques to enhance the pump power conversion efficiency in Ytterbium-doped fibers should be explored as a future work direction. 

## Figures and Tables

**Table 1:** Different symbols used in Equations (1)–(8).

Symbol	Description
$A_{ij}$	Spontaneous decay rates between i and j levels
$W_{ij}$	Stimulated emission rates between i and j levels
$R_{ij}$	Pumping rates between i and j levels
$n_{1}$, $n_{2}$	Population densities at ground and excited states
$n_{t}$	Total population density
$I_{p}$, $I_{s}$	Pump and signal intensities
$h\upsilon_{p}$, $h\upsilon_{s}$	Pump and signal photon energies
$\sigma_{12}^{p}$, $\sigma_{21}^{p}$	Pump absorption and emission cross sections
$\sigma_{12}^{s}$, $\sigma_{21}^{s}$	Signal absorption and emission cross sections
*z*	Longitudinal position along fiber
$P_{p}$, $P_{s}$	Pump and signal powers
$\eta_{p}$, $\eta_{s}$	Pump and signal overlap factors

**Table 2:** Important simulation parameters and their values.

Parameter	Value
Wavelength of P-1	0.92 μm
Wavelength of P-2	0.98 μm
Power of P-1	1 W
Power of P-2	4 W
Length of Y-1	1 m
Yb${}^{3+}$ concentration in Y-1	50 × 10${}^{24}$ m${}^{-3}$
Core radius of Y-1 and Y-2	3.4 μm
Doping radius of Y-1 and Y-2	2.4 μm
Cladding radius of Y-1 and Y-2	62.5 μm
Numerical aperture of core of Y-1 and Y-2	0.2
Numerical aperture of cladding of Y-1 and Y-2	0.5
Signal attenuation	0.1 dB
Pump attenuation	0.15 dB
Temperature	300 K

**Table 3:** Comparison of the important results of the proposed work with results of the past related studies.

Study	Pumping Stages	Pumps	Gain	Output Power	NF	PCE
[8]	Single	1	-	100 mW	-	11.5%
[7]	Single	2	45 dB	-	-	
[5]	Dual	2	21 dB	-	-	
[9]	Dual	2	25.5 dB	-	-	
[12]	Single	1	-	6.7 W	-	38%
[13]	Single with dual pass	1	25 dB	-	3.5 dB	-
[27]	Single	1	32 dB	0.2 W	4 dB	-
Proposed	Dual	2	62.5 dB	3.5 W	4 dB	60.5%

## Abbreviations

The following abbreviations are used in this manuscript:

YDFA	Ytterbium-doped fiber amplifier
NF	Noise figure
EDFA	Erbium-doped fiber amplifier
CW	Continuous wave
MOPA	Master oscillator power amplifier
FCPA	Fiber chirped power amplifier
YDF	Ytterbium-doped fiber
ASE	Amplified spontaneous emission
OPM	Optical power meter
OSA	Optical spectrum analyzer
OSNR	Optical signal to noise ratio
